# Differential Impact of Plant Secondary Metabolites on the Soil Microbiota

**DOI:** 10.3389/fmicb.2021.666010

**Published:** 2021-05-28

**Authors:** Vadim Schütz, Katharina Frindte, Jiaxin Cui, Pengfan Zhang, Stéphane Hacquard, Paul Schulze-Lefert, Claudia Knief, Margot Schulz, Peter Dörmann

**Affiliations:** ^1^Institute of Molecular Physiology and Biotechnology of Plants, Bonn, Germany; ^2^Institute of Crop Science and Resource Conservation, Molecular Biology of the Rhizosphere, Bonn, Germany; ^3^Max Planck Institute for Plant Breeding Research, Cologne, Germany

**Keywords:** bacteria, soil, microbiota, benzoxazinoid, benzoxazolinone, gramine, quercetin

## Abstract

Plant metabolites can shape the microbial community composition in the soil. Two indole metabolites, benzoxazolinone (BOA) and gramine, produced by different Gramineae species, and quercetin, a flavonoid synthesized by many dicot species, were studied for their impacts on the community structure of field soil bacteria. The three plant metabolites were directly added to agricultural soil over a period of 28 days. Alterations in bacterial composition were monitored by next generation sequencing of 16S *rRNA* gene PCR products and phospholipid fatty acid analysis. Treatment of the soil with the plant metabolites altered the community composition from phylum to amplicon sequence variant (ASV) level. Alpha diversity was significantly reduced by BOA or quercetin, but not by gramine. BOA treatment caused a decrease of the relative abundance of 11 ASVs, while only 10 ASVs were increased. Gramine or quercetin treatment resulted in the increase in relative abundance of many more ASVs (33 or 38, respectively), most of them belonging to the Proteobacteria. Isolation and characterization of cultivable bacteria indicated an enrichment in *Pseudarthrobacter* or *Pseudomonas* strains under BOA/quercetin or BOA/gramine treatments, respectively. Therefore, the effects of the treatments on soil bacteria were characteristic for each metabolite, with BOA exerting a predominantly inhibitory effect, with only few genera being able to proliferate, while gramine and quercetin caused the proliferation of many potentially beneficial strains. As a consequence, BOA or gramine biosynthesis, which have evolved in different barley species, is accompanied with the association of distinct bacterial communities in the soil, presumably after mutual adaptation during evolution.

## Introduction

Plants establish their specific microbial environment, starting with proliferation of the endophytic microbiota, and including microorganisms attracted from the soil after germination (Berg and Raaijmakers, 2018). Root exudates are crucial for modulating the microbial species composition in the rhizosphere to improve plant growth and the competitiveness with other organisms (Andreo et al., 1984; La Hovary et al., 2016; Suseela et al., 2016; Hu et al., 2018; Kudjordjie et al., 2019). Metabolites and their derivatives exuded from the roots likely contribute to the adjustment of the plant microbiota and modulate its functional diversity. For example, salicylic acid exuded from willow tree roots modulates the microbiota and alters microbial composition in the rhizosphere (Schmidt et al., 2000). Moreover, a considerable amount of allelopathic and microbiota-modifying metabolites is released from rotting plant material, and, in appropriate agricultural culture systems, also from mulches, thereby influencing the soil microbial communities in the entire agricultural ecosystem. For example, the allelopathic properties of benzoxazinoids (BXs) are long known and have been intensively investigated, including the occurrence of microbial degradation products in the soil (Reberg-Horton et al., 2005; Tabaglio et al., 2008; Schulz et al., 2013). Furthermore, allelopathic properties have been described for gramine and quercetin (Szwed et al., 2019; Maver et al., 2020).

Benzoxazinoids are a class of indole-derived metabolites with allelochemical activity synthesized in Poaceae, including some *Hordeum* species (e.g., *Hordeum brachyantherum*, *Hordeum flexuosum*, *Hordeum lechleri*) (Sicker et al., 2000; Grün et al., 2005). They are typically synthesized in young plants and have allelopathic, phytotoxic and other biocidal properties including antinutritional functions in herbivores (Wouters et al., 2016). BXs are unstable in soil, where they are rapidly converted into benzoxazolin-2(3H)-one (BOA) and 6-methoxy-2-benzoxazolinone (MBOA), which are detectable in the soil for up to 12 weeks (Fomsgaard et al., 2004; Macías et al., 2005). Because of their phytotoxicity, these compounds have been selected for weed control in agriculture (Schulz et al., 2013). BOA can be degraded or detoxified by a few fungi and bacteria (Schulz et al., 2013, 2017). Changes in the structure of the rhizosphere microbiota of maize wild type and the BX-deficient *bx1* mutant plants have been studied. Cultivation of WT or *bx1* plants resulted in a shift in bacterial and fungal species composition, which affected growth of the following plant generation (Hu et al., 2018). Several bacterial and few fungal operational taxonomic units in the rhizosphere of maize were influenced by different *bx* (*bx1, bx2, bx6*) mutations (Cotton et al., 2019). Growth of the same three maize mutants led to a decline in fungal and bacterial species richness in the rhizosphere (Kudjordjie et al., 2019). While young maize plants mostly exude methoxylated forms of BXs (e.g., 2,4-dihydroxy-7-methoxy-1,4-benzoxazin-3-one, DIMBOA), which are converted into MBOA in the soil, the leaves of old plants lack DIMBOA and instead accumulate 2,4-dihydroxy-1,4-benzoxazin-3-one (DIBOA), which is converted into BOA (Köhler et al., 2015). The phytotoxic and allelopathic activities of BXs and their effects in weed control have so far been mostly studied with BOA in laboratory experiments. Field studies for weed control were performed with rye mulches, which contain DIBOA (which is converted into BOA) (Schulz et al., 2013). BOA is not only found in rye, but also in wheat and can even be released from BX-containing dicots (Reberg-Horton et al., 2005; Rice et al., 2005; Understrup et al., 2005). However, the effects of BOA on microbiota composition and species diversity in the soil remain unclear.

Some Poaceae produce gramine as an additional indole-derived metabolite. Gramine is one of the most important allelochemicals in those barley cultivars that do not contain BXs (*Hordeum vulgare* L., e.g., genotypes Lina, Osiris) (Larsson et al., 2006; Kokubo et al., 2017). Gramine is also found in other plants, e.g., lupines (*Lupinus luteus* L.). It affects germination and growth of oat, rye, and wheat (Bravo et al., 2010). For allelopathic effects, gramine has to be released from rotting plant material, because in contrast to BXs, gramine seems to be absent from root exudates. Gramine also inhibits growth of cyanobacteria and other bacteria (Laue et al., 2014; Popp et al., 2016). The detoxification pathway of gramine in aphids (*Sitobion avenae*) involves carboxylesterase and glutathione S-transferase activities (Cai et al., 2004), but bacterial or plant detoxification strategies are unclear. Barley cultivars produce only one of the two metabolites, BX or gramine. Results of this study show that BX and gramine have distinct effects on the soil microbiota.

Flavonoids are polyphenols that occur widespread in plants, in particular in seeds and root exudates, with quercetin representing one of the most abundant flavonoids (Mathesius, 2018). Flavonoids are involved in the regulation of nodulation in legumes, they exert antioxidant and antimicrobial activities, and many of them have allelopathic properties. Several flavonoids have a short lifetime while others persist in the soil, depending on the chemical structures, such as the number of functional groups, physicochemical characteristics of the soil and the rate of microbial degradation (Chaaban et al., 2017). Many Fabaceae like *Lotus japonicus* contain quercetin as the main flavonoid (Suzuki et al., 2008). Several microorganisms such as *Rhizobium*, *Agrobacterium*, *Pseudomonas*, *Bacillus*, and *Rhodococcus* species degrade flavonoids including quercetin (Pillai and Swarup, 2002). *Rhizobium* species detoxify flavonoids and isoflavonoids via C ring fission, which results in phenolic compounds like protocatechuic acid. The prooxidant activity of protocatechuic acid can cause oxidative stress and death of other microorganisms. Protocatechuic acid is also responsible for the indirect allelopathic effects of catechin, which is e.g., exuded by roots of *Rhododendron formosanum* (Wang et al., 2013). The rhizosphere of *R. formosanum* is rich in microbial taxa that use catechin as carbon source, including *Pseudomonas*, *Herbaspirillum*, and *Burkholderia*. Therefore, flavonoid breakdown products might be the true modifiers of microbial biodiversity and influence the abundance of defined species. A recent study showed that flavones lead to the enrichment of members of the Oxalobacteraceae in the rhizosphere of maize which promote growth and nitrogen acquisition (Yu et al., 2021).

To study the effects of selected plant-root derived metabolites on soil microbial communities in comparison, native agricultural soil was exposed to BOA, gramine or quercetin, using concentrations that occur under natural conditions (Barnes and Putnam, 1987; Reberg-Horton et al., 2005; Carlsen et al., 2012; Wang et al., 2018; Maver et al., 2020). We selected BOA and gramine, which are two indole metabolites produced in a mutually exclusive manner by Poaceae species, to study their impact on the soil microbiota (Larsson et al., 2006). In addition, we chose to study the effects of quercetin on the soil microbiota with the aim to assess how specific the changes in the soil microbial community introduced by the two indole metabolites are. Bacterial 16S *rRNA* gene-based community profiling in combination with phospholipid fatty acid analyses revealed that the effects on bacterial diversity and community composition of BOA and gramine were very different, while the effects of gramine and quercetin were related. Microorganisms were retrieved from the soil after application of the aforementioned plant compounds with the aim of unraveling whether bacterial genera that increased in abundance according to 16S *rRNA* gene community profiling, can be enriched and isolated for future studies.

## Materials and Methods

### Incubation of Soil With Secondary Metabolites

The Cologne agricultural soil was obtained from a local site (Bai et al., 2015). The nutrient composition is provided in Harbort et al. (2020). The soil is slightly acidic (pH 6.5), with a water content of 12.0 ± 1.1%, and it was stored at 4°C for 7–14 days after collecting it from the field before starting the experiments. The soil was sieved (2 mm mesh) and filled into open pots (10 cm diameter, 375 ml volume) which were placed onto Petri dishes. Because none of the metabolites are easily soluble in water, the compounds were added to the soil in solid form. An amount of 10 μmol of solid BOA (1.4 mg; Fluka Chemika), gramine (1.8 mg; Sigma-Aldrich) or quercetin (3.4 mg; ABCR, Karlsruhe, Germany) was weighed and mixed with 300 g of soil with a clean spatula. Next, 50 ml of water were added to achieve a water content of ∼24.5%, equivalent to ∼60% of the maximal water retention capacity (which is at ∼40% water content). The treatment was repeated every second day over a period of 28 days, and the water content maintained at ∼24.5%. In total, 140 μmol of metabolites were added to each pot equivalent to ∼1.6 mM (based on a water content of 24.5%). Control soil was watered with germ-free water without the addition of metabolites. The pots were covered with transparent plastic covers with holes and incubated in a growth chamber under a 16 h light/8 h dark cycle with 160 μmol/m^2^/s of light, at 21°C and 55% humidity. Four replicate pots were prepared per treatment. Soil samples (0.5 g each) were removed with a spatula every seven days until day 28, and placed into a Lysing Matrix E tube for DNA extraction (FastDNA SPIN Kit for Soil, MP Biomedicals, Solon, United States, see below).

### Extraction and Measurement of Metabolites From Soil

After the treatment with 10 μmol BOA, gramine or quercetin, the metabolites were extracted from 300 g of soil with methanol containing 0.1% formic acid. The soil/methanol mixture was sonicated for 5 min followed by an incubation under vigorous shaking for 30 min. The slurry was centrifuged at 4,000 × *g* for 10 min. The organic phase was harvested and concentrated using a rotary evaporator. The metabolites were analyzed by HPLC with a diode array detector (Shimadzu) with an analytical reversed phase C18 column (Nucleodur, Macherey-Nagel, Germany) as described by Schulz et al. (2018).

### Phospholipid Fatty Acid Analysis

Lipids were extracted from the soil with chloroform/methanol. Phospholipids were isolated by solid phase extraction, fatty acids were converted into their methyl derivatives in the presence of the internal standard tridecanoic acid (13:0) and quantified by gas chromatography-mass spectrometry as described (Siebers et al., 2018).

### Isolation and Identification of Cultivable Bacteria From Soil

Bacterial strains were isolated from the soil after 28 days of treatment with BOA, gramine or quercetin. The soil from one representative pot each (300 g, 12% water content) derived from the control or one of the metabolite treatments (after 28 days) was mixed with 300 ml of sterile water. Soil suspensions were mixed and centrifuged at 2,000 × *g* for 15 min. Aliquots (50 μL) of a 10-fold dilution series were plated on four different media for the growth of bacterial or fungal isolates: TSB medium (17 g/L casein peptone, 3 g/L soya peptone, 5 g/L NaCl, 2.5 g/L K_2_HPO_4_, 2.5 g/L glucose, 15 g/L agar), YPD medium (10 g/L yeast extract, 20 g/L bacto peptone, 20 g/L glucose, 20 g/L agar), TSM medium (1 g/L K_2_HPO_4_, 0.2 g/L MgSO_4_, 0.1 g/L CaCl_2_, 0.1 g/L NaCl, 0.002 g/L FeCl_3_, 0.5 g/L KNO_3_, 0.5 g/L asparagine, 1 g/L mannitol, 15 g/L agar), malt medium (15 g/L malt extract, 8 g/L yeast extract, 5 g/L glucose, 5 g/L fructose, 10 g/L agar), Czapek medium (2 g/L NaNO_3_, 0.5 g/L MgSO_4_⋅7H_2_O, 1 g/L K_2_HPO_4_, 0.5 g/L KCl, 0.01 g/L FeSO_4_, 30 g/L sucrose, 13 g/L Bacto agar) and PGA medium (10 g/L peptone, 20 g/L glucose, 20 g/L agar). Plates were incubated for several days at 28°C. Upon isolation, colonies were picked for PCR amplification of the bacterial 16S *rRNA* gene using primers 799F and 1192R which target the V4-V7 region (for PCR conditions, see section “Isolation and Identification of Cultivable Bacteria From Soil”). PCR products were purified by excising the 500 bp bands after electrophoresis in 1.2% agarose gels. DNA was eluted using the NucleoSpin Gel and PCR Clean-up Kit (Macherey-Nagel). Obtained isolates were stored at −80°C in microbanks. The 16S rRNA gene sequences of the isolated strains were quality checked and trimmed before they were aligned using the SINA aligner v1.2.11 (Pruesse et al., 2012) and imported into the SSU Ref NR 99 138.1 database. The alignment was manually controlled and a phylogenetic tree was calculated using the maximum-likelihood algorithm PhyML in ARB (Ludwig et al., 2004).

### Bacterial Community Profiling by 16S *rRNA* Amplicon Sequencing

Total DNA was extracted from the soil using the FastDNA SPIN Kit for Soil (MP Biomedicals, Solon, United States). To the soil in the Lysing Matrix E tube, sodium phosphate buffer and MT buffer were added, and it was homogenized with 2 × 30 s rotations at 6,000 rpm in a Precellys tissue homogenizer (Bertin, Frankfurt a. M., Germany). Further extraction steps were performed as described in the manufacturer’s protocol. Genomic DNA from soil was eluted in 60 μL of nuclease free water and purified twice using Agencourt MPure XP beads (Beckman-Coulter, Krefeld, Germany). The purified genomic DNA was used in a two-step PCR procedure to amplify the V4-V7 region of the bacterial 16S *rRN*A gene (primers 799F–1192R, Supplementary Table 1) (Bodenhausen et al., 2013). In the first step, the gene fragment was amplified for each sample in triplicates in a 25 μL reaction volume (Agler et al., 2016). Each reaction contained 0.2 μM of each primer, GoTaq Reaction Buffer (1.5 mM MgCl_2_), PCR Nucleotide Mix (0.2 mM each dNTP), 1.25 U GoTaq G2 DNA polymerase (Promega, Walldorf, Germany), 0.3% bovine serum albumin, 4 ng template DNA and nuclease-free water. The first PCR amplification was performed as follows: 2 min at 94°C; 25 cycles of 15 s at 94°C, 25 s at 55°C, 45 s at 72°C. The products were purified by electrophoresis in 1.2% agarose gels. The bands at 500 bp were excised and the DNA purified using the NucleoSpin Gel and PCR Clean-up kit (Macherey-Nagel, Düren, Germany). DNA was finally eluted in 20 μL nuclease free water. The purified products were used for a second PCR, which was performed in the same way but with only 15 cycles. In the second PCR, barcoded primers containing Illumina adaptors (B5-F; B5-1 to B5-64; Supplementary Table 1) were used. The PCR products were also purified by electrophoresis (see above). The DNA concentration of the purified PCR products was determined by fluorometry (QuantiFluor ONE Dye, Quantus Fluorometer, Promega, Walldorf, Germany), and a sequencing library was created by pooling 30 ng of each amplicon. The library was purified twice with Agencourt MPure XP beads.

### Illumina Sequencing

A total of 100 ng of DNA the sequencing library were used for 2 × 300 bp paired-end Illumina sequencing on a MiSeq sequencer (Illumina, Berlin, Germany). Paired-end Illumina sequencing was performed by using custom sequencing primers (for details see: Durán et al., 2018; Supplementary Table 1). Raw sequence data were merged with Flash2 (Magoè and Salzberg, 2011) which also results in the removal of reads shorter than 300 bp. Further analyses were performed in QIIME2 (Version 2019.4) to obtain an amplicon sequence variant (ASV) table (Bolyen et al., 2019). The sequences were thereby denoised by using DADA2 which includes a strict quality control by discarding reads with ambiguous bases, singletons and chimera. Chimeras were additionally removed by using Uchime. ASVs were taxonomically classified with the SILVA database 138 by using the naïve Bayesian algorithm provided in QIIME2, using the recently revised bacterial taxonomy system. Sequences assigned to chloroplasts and mitochondria were removed from the final ASV table.

### Statistics

The ASV table was rarefied to the lowest number of reads (7,100 reads) to obtain equal read numbers for all samples for alpha diversity analysis. Alpha-diversity indices (Shannon, Faith PD, and Pilou’s evenness) were calculated in QIIME2 (version 2020.11). Overall differences between groups of samples were assessed using non-parametric a Kruskal-Wallis test (for treatment) and a Friedman test (for the time series). To investigate for differences due to the factors treatment (control, BOA, gramine, and quercetin), additional Dunn tests were applied for individual comparisons (package FSA in R). Pairwise Friedman tests were used to explore the difference in the time series (t7, t14, t21, t26) in SPSS 25. A Bonferroni-Holm correction was performed to correct for multiple comparisons.

A principle component (PCA) plot based on clr normalized Aitchison distance matrices was built using the DEICODE tool in QIIME2 (Martino et al., 2019). To test for significant differences in the bacterial community composition in dependence on treatment and time, Permutational ANOVA (ADONIS) was performed using the clr-normalized Aitchison distance matrix.

Responsive taxa significantly enriched in relative abundance due to sample treatment and time were identified at ASV levels using ANCOM implemented in QIIME2 (Mandal et al., 2015). A heatmap was generated in R using the packages pheatmap and dplyr (Kolde, 2019; Wickham et al., 2020), which shows the preferential occurrence of the most abundant significantly impacted ASVs (>1% relative abundance) in dependence on treatment and time point. Dendrograms were calculated using the WPGMA clustering algorithm based on an Euclidean distance matrix derived from relative ASV abundance. Since ANCOM cannot distinguish between which factors the differences occur when there are more than two factors, we complemented the analyses by using STAMP (Parks et al., 2014) in addition to the comparison of median values provided by ANCOM. Therefore, we performed a Kruskal-Wallis test in STAMP with additional Tukey-Kramer Post-hoc tests corrected via a strict Bonferroni procedure to investigate between which treatments the significant differences occurred for the ASVs found significantly impacted by ANCOM.

## Results

### Treatment of Agricultural Soil With BOA, Gramine, or Quercetin

We incubated agricultural soil in open pots with the addition of one of the three plant metabolites, BOA, gramine, or quercetin. The metabolites (10 μmol) were added in a dry form and mixed into 300 g of soil. This treatment was repeated every other day to maintain a certain level of metabolites in the soil over the period of 28 days. The contents of BOA, gramine or quercetin were measured by HPLC to determine the amounts of metabolites retrievable from the soil after 2 days of incubation. HPLC analysis revealed that the amounts of the metabolites were strongly decreased after 2 days. At the same time, low amounts of additional compounds derived from microbial degradation of BOA, gramine or quercetin were found. After two days, only 1.50 ± 0.26 μmol BOA were found in 300 g of the soil. In addition, one major degradation product was found (0.89 ± 0.17 μmol) which was identified as 2-aminophenoxazinone (APO), a known microbial degradation product of BOA. In the gramine treated soils, 0.09 ± 0.03 μmol of gramine and two unknown degradation products were detected in 300 g of soil after 2 days of treatment. The amount of quercetin was below the detection limit 2 days after the treatment of the soil, while several degradation products were found. The strong decrease in metabolites two days after treatment could be due to absorption to soil particles or to degradation by the soil microbiota. To determine the proportion of the metabolites trapped by binding to soil particles, BOA, gramine or quercetin were mixed into the soil and immediately extracted for subsequent HPLC analysis (0 day samples in Supplementary Figure 1). In this experiment, 11.42 ± 1.87 μmol of BOA were retrieved, but only 0.35 ± 0.05 μmol of gramine, while no quercetin could be extracted. Therefore, BOA is not bound to soil particles, but gramine and quercetin are strongly bound to the soil particles, affecting their extraction from soil.

### Phospholipid Fatty Acid Measurements of Soil Samples

**FIGURE 1:**
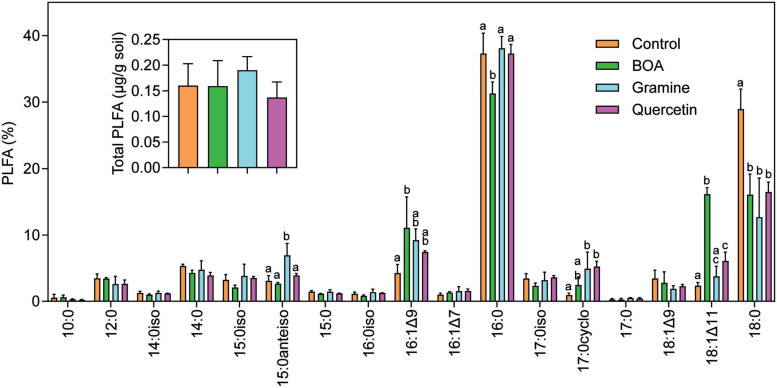
Phospholipid fatty acid (PLFA) analysis of soil samples. Soil samples harvested after 28 days of treatment with BOA, gramine or quercetin were extracted and phospholipid fatty acids determined by GC-MS. The inset shows total PLFA in μg/g of dried soil. 10:0, decanoic acid; 12:0, lauric acid; 14:0iso, 11-methyl-tridecanoic acid; 15:0iso, 13-methyl-myristic acid; 15:0anteiso, 12-methyl-myristic acid; 16:0iso, 13-methyl-pentadecanoic acid; 16:1Δ9, palmitoleic acid; 16:1Δ7, Δ7-hexadecenoic acid; 16:0, palmitic acid; 17:0iso, 15-methyl-palmitic acid; 17:0cyclo, 7,8-cyclopropane-palmitic acid; 18:1Δ9, oleic acid; 18:1Δ11, vaccenic acid; 18:0, stearic acid. (ANOVA, *post hoc* Tukey; *p* < 0.05; *n* = 3; mean ± SD; different letters indicate significant differences).

The phospholipid fatty acid (PLFA) content and composition was recorded to assess the impact of the metabolites on the total amount and composition of soil microbiota. Thus, phospholipids were extracted and purified from the soil after 28 days of incubation, and PLFA were measured by GC-MS after conversion into methyl esters. The total amounts of PLFA, which can serve as a measure for the total amount of microbial biomass in soil, were ∼0.15 μg/g soil in all samples without significant differences (Figure 1). Therefore, the total amounts of microorganisms in the soil were not affected after the treatment with BOA, gramine or quercetin in comparison with the control. The most abundant PLFA detected in the soil samples were the saturated fatty acids 16:0 and 18:0. Furthermore, odd chain (e.g., 15:0, 17:0), methyl-branched (e.g., 15:0iso, 15:0anteiso), monounsaturated (e.g., 16:1Δ9, 16:1Δ7, 18:1Δ11) and cyclopropane (17:0cyclo) fatty acids were identified. Odd chain, methyl-branched and monounsaturated fatty acids are mainly found in bacteria, while cyclopropane fatty acids are produced in bacteria after stress (Kaneda, 1991; Alvarez-Ordóñez et al., 2008). Indeed, we observed increases in the amount of the cyclopropane fatty acid 17:0cyclo indicating that the bacteria were subject to stress after metabolite supplementation. Furthermore, changes in specific fatty acids were observed between the different treatments and in comparison with the control. All treatments caused a decrease in 18:0. Under BOA treatment, 16:1Δ9 and 18:1Δ11 were increased, accompanied with a specific decrease in 16:0. During gramine treatment, 15:0anteiso was increased, and after quercetin treatment, 18:1Δ11 was elevated. These changes in fatty acids point to alterations in the relative abundances of specific bacterial taxa dependent on the metabolite treatment. Fatty acids typically found in fungi like oleic acid (18:1Δ9) or diunsaturated fatty acids (e.g.18:2Δ9,12), or in cyanobacteria/algae (triunsaturated fatty acids, 16:3Δ7,10,13; 18:3Δ9,12,15) (Frostegård and Bååth, 1996; Ibekwe and Kennedy, 1998; Kaiser et al., 2010), were of low abundance or absent from the soil. Therefore, the soil was dominated by the presence of bacterial biomass, and very low amounts of fungal biomass.

### Responses of the Soil Microbiota to Plant Metabolites

The microbiota structure in the soil during the metabolite treatment was analyzed by amplicon sequencing of extracted DNA. The fragments amplified by two rounds of the bacterial 16S *rRN*A gene (V4-V7 region, primers 799F, 1192R) or of the fungal ITS gene region (primers ITS1F, ITS2) (Agler et al., 2016) were separated and isolated by agarose gel electrophoresis. While considerable amounts of bacterial DNA were amplified from the soil samples and analyzed by next generation sequencing (NGS), the fungal DNA fragments were barely detectable, and no sequences were obtained after NGS. Therefore, the samples of the soil were largely devoid of fungi, and only the bacterial sequences were analyzed in the following.

**FIGURE 2:**
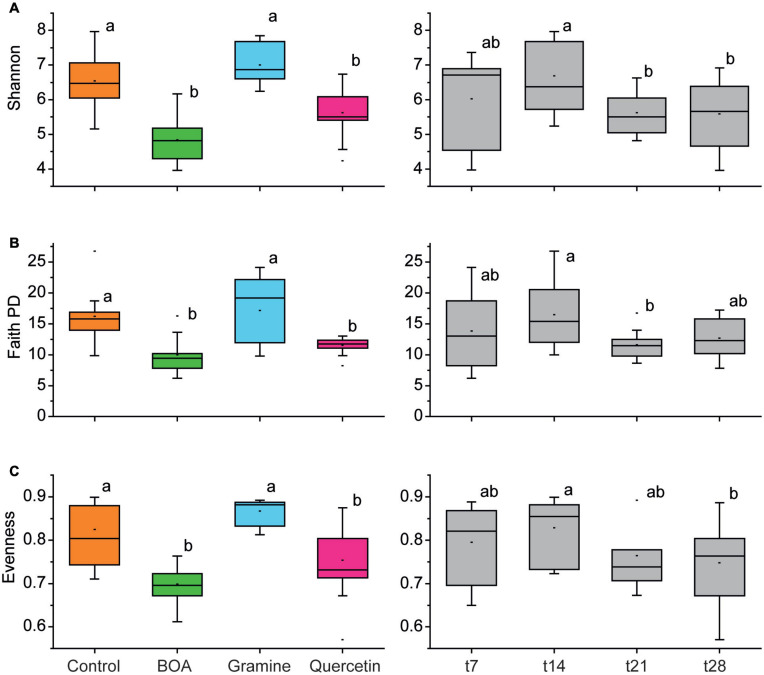
Box plots showing differences in alpha-diversity measures of the soil bacterial communities in dependence on treatments with different plant metabolites and over time. Boxplots are shown for the Shannon index **(A)**, Faith PD index **(B)**, and Evenness index **(C)**. All time points of each metabolite treatment **(left column)**, and all treatments of a given time point were aggregated (right columns); *n* = 4 (each box represents 16 data points). Significant differences are indicated by lower-case letters according to pairwise Mann–Whitney (left panels) or Friedman tests (right panels) with respective correction for multiple comparison.

Different alpha diversity indices (Shannon, Faith PD and Pilou‘s Evenness) as measures for biodiversity, phylogenetic diversity and equal distribution of species in the bacterial community, respectively, were calculated (Figure 2). The alpha diversity indices were aggregated for all time points of a given treatment (left panels in Figure 2), or for all treatments of a given time point (right panels in Figure 2). All alpha diversity indices showed a significant reduction after BOA or quercetin treatment compared to the control. In contrast, alpha diversity indices of gramine-treated samples were not significantly different from the control. The alpha diversity indices remained largely similar over time. Only the time points t21 and partly t28 showed a small and in part significant decrease compared with t14.

**FIGURE 3:**
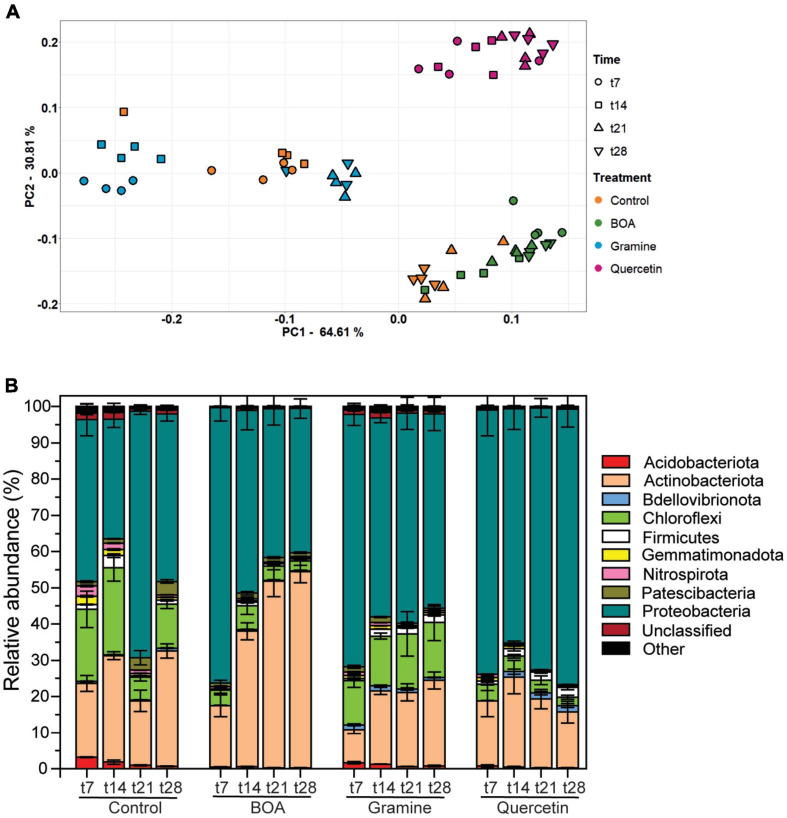
Changes in the soil bacterial community structure after the treatment with BOA, gramine or quercetin. **(A)** Differences in bacterial community structure between samples are illustrated in principle component (PCA) plots. The time points are shown by different symbols, and the color code depicts the different treatments. Each measurement is represented by 4 replicates. **(B)** Relative abundance of bacterial phyla in the soil of the control or after treatment with BOA, gramine or quercetin. Low abundant groups with <2% of the total reads are summarized as “Other”. (*n* = 4, mean ± SD).

Changes in the bacterial community structure were evaluated in a PCA plot (Figure 3A). A clustering according to treatment was evident, while temporal differences explained less of the variation in community composition between samples. These observations were confirmed by ADONIS with higher R^2^-values for treatment then for time points (treatment, R^2^ = 0.746, *P* = 0.001; time point, R^2^ = 0.110, *P* = 0.001; interaction effect, treatment × time point, R^2^ = 0.100, *P* = 0.001). The samples treated with BOA, gramine or quercetin were located in separate clusters. For the control samples, the early time points (t7, t14) were separated from the late ones (t21, t28), which partially overlapped with the BOA treated samples. In addition, the early time points (t7, t14 of gramine treated samples were separated from the late time points (t21, t28), which partially overlapped with the early time points of the control. This indicates that the bacterial community structure was strongly altered by quercetin treatment while the effects of BOA and gramine were less pronounced compared with the control.

### Bacterial Taxa Are Differentially Affected by Plant Metabolites

The phyla of Actinobacteriota, Chloroflexi, and Proteobacteria were most abundant in the control soil samples with mean relative abundances of 31.9, 12.1, and 46.2% at the time point t28, respectively, while Acidobacteriota, Bdellovibrionota, Firmicutes, Gemmatimonadota, Nitrospirota, Patescibacteria, unclassified bacteria and others were much less abundant (all <2%, Figure 3B). Exposure to the plant metabolites revealed changes in taxonomic composition. Incubation with BOA resulted in an increase in the relative abundances of Actinobacteriota up to 54.2% with a concomitant decrease in Proteobacteria (39.9%)and Chloroflexi (2.7%) at time point t28 compared to the control treatment. In contrast, the exposure to gramine did not affect the relative abundance of Actinobacteriota (23.7%) at t28 compared to the control treatment, while Proteobacteria were more abundant with 53.5% (Figure 3B). Quercetin treatment resulted in lower relative abundances of Chloroflexi (2.2%), and higher percentages of Proteobacteria (76.0%), and Bdellovibrionota (1.8%) at t28.

**FIGURE 4:**
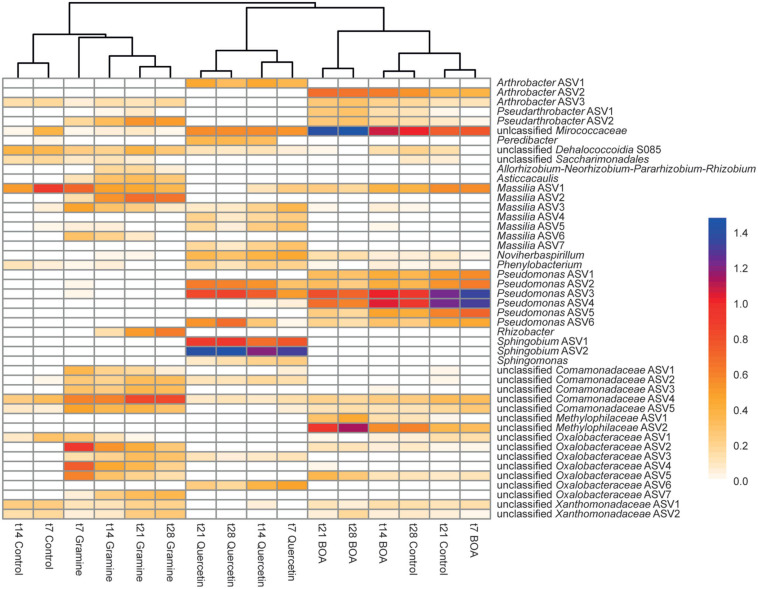
Heatmap showing differences in relative abundances of bacterial ASVs in soil after application of different plant metabolites over time. Differences in relative abundance (log10-transformed) as determined by ANCOM analysis are color-coded with blue colors indicating high relative abundances, and yellow colors indicating low abundances. Only ASVs with a relative abundance >1% under at least one condition are shown. For a full list of ASVs showing significant differences in relative abundance see Supplementary Table 2. The dendrogram was derived from WPGMA clustering of an Euclidean distance matrix.

The treatment with the three plant-derived metabolites also resulted in characteristic changes on the ASV level. Ninety-six bacterial ASVs showed significant differences in relative abundance in response to the treatment with BOA, gramine or quercetin (all time points aggregated) (Supplementary Table 2). The ASVs with relative abundances >1.0% at any time point in the control or one of the three treatments were included in a heatmap showing the changes in relative abundances over time (Figure 4), and the time courses of selected ASVs are shown in Supplementary Figure 2. The clustering of the samples in the heatmap reflected the PCA results with a separation of samples according the three treatments with the plant metabolites (Figure 3A). In agreement with the overlap of the data points in the PCA plot, early time points of the control clustered most closely to the gramine treatment, while late control time points were most similar to the BOA treatment.

**FIGURE 5:**
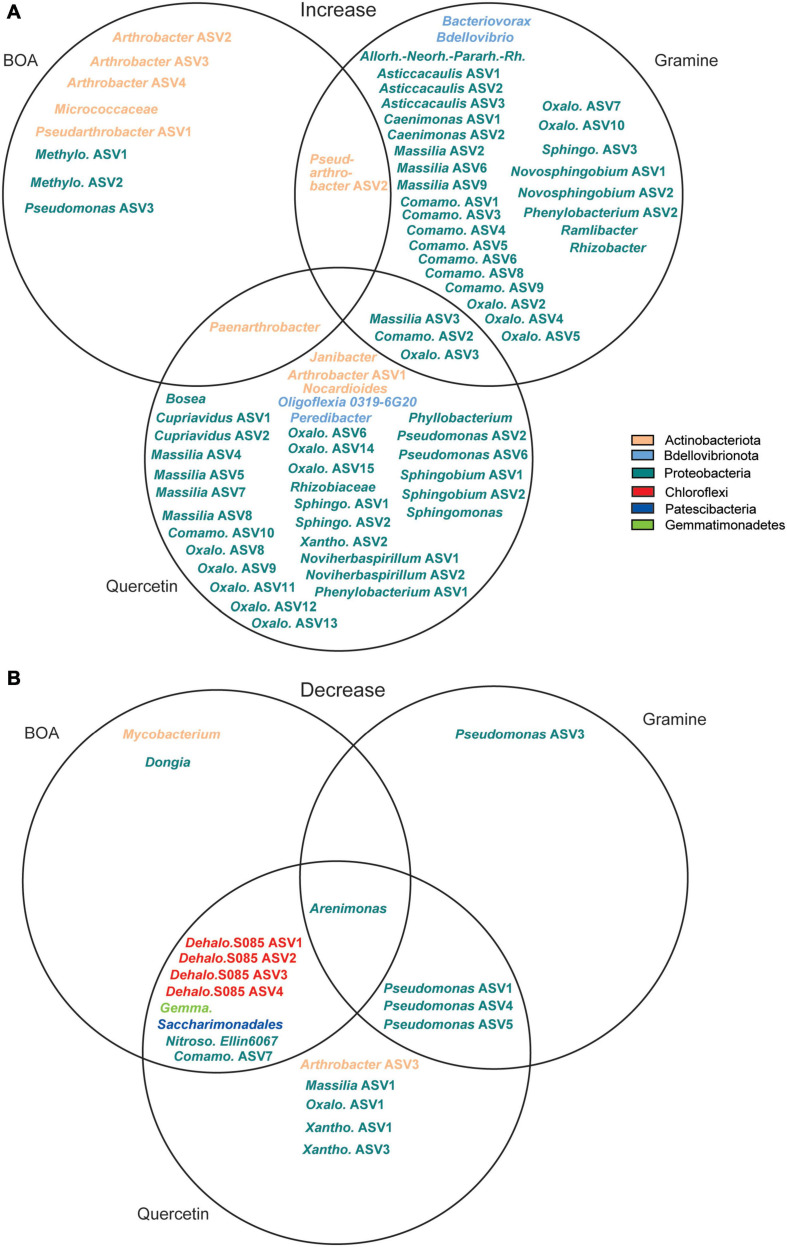
Venn diagrams showing ASVs with **(A)** increased or **(B)** decreased relative abundance after treatment with BOA, gramine or quercetin in comparison to the control. Different colors indicate the assignment to the different phyla. Allorh.-Neorh.-Pararh.-Rh., *Allorhizobium-Neorhizobium-Pararhizobium-Rhizobium*; Comano., Comamonadaceae; Dehalo., Dehalococcoidia; Gemma., Gemmatimonadaceae; Meth., Methylophilaceae; Nitroso., Nitrosomonadaceae; Oxalo., Oxalobacteraceae; Sphingo., Sphingomonadaceae; Xanth., Xanthomonadaceae.

ASVs with a significant increase or decrease in relative abundance after treatment with the metabolites are presented in two Venn diagrams (Figure 5). Seven ASVs of Actinobacteriota (*Arthrobacter*, *Pseudarthrobacter*, *Paenarthrobacter*, Micrococcaceae) and three of Proteobacteria (*Pseudomonas*, Methylophilaceae), showed an increase in relative abundance after BOA treatment. The relative abundance of *Pseudarthrobacter* ASV2 was also increased after gramine treatment, while *Paenarthrobacter* accumulated in addition after quercetin treatment. Gramine treatment specifically caused the accumulation of 31 ASVs of Proteobacteria, two ASVs of Bdellovibrionota, and three additional Proteobacteria ASVs increased in abundance during gramine or quercetin treatment. Similarly, the ASVs that specifically increased in relative abundance upon quercetin treatment were mostly Proteobacteria (29 ASVs), though different ones than upon gramine treatment, besides Actinobacteriota (3 ASVs) and Bdellovibrionota (1 ASV). Several ASVs decreased in relative abundance during the metabolite treatments, some even below the detection limit, after application of either of the three metabolites. While the number of ASVs that decreased in abundance was similar to the ones that increased for BOA treatment (11 vs. 10 ASVs), much less ASVs were decreased than increased during gramine or quercetin treatment. Several ASVs, e.g., *Massilia* ASV3, showed a decrease in relative abundance after the treatment with one or more metabolites, indicating that their growth was limited by the plant metabolites in comparison with other bacteria (Figure 4 and Supplementary Figure 2). Other ASVs like *Arthrobacter* ASV2 or *Pseudarthrobacter* ASV1, showed a gradual increase in relative abundance over time, but were also increased in the control experiment, albeit to a lower extent, suggesting that these bacteria are not affected by the metabolites. A few ASVs like *Arthrobacter* ASV1 or ASV2, showed a maximal relative abundance at intermediate time points, i.e., after 14 and 21 days of treatment (Supplementary Figure 2A).

Of the 96 ASVs that were significantly changed upon treatment with the plant metabolites, most were of very low abundance, and only 10 were showed a relative abundance of >2% under at least one condition (Supplementary Table 2 and Figure 2). Two *Massilia* ASVs (ASV1, ASV2), one unclassified Comamonadaceae ASV (ASV4) and two unclassified Oxalobacteraceae ASVs (ASV1, ASV2) showed abundances > 2% after gramine addition. Furthermore, two *Arthrobacter* ASVs (ASV2, ASV5) and one unclassified Methylophilaceae ASV accumulated to >2% under BOA treatment (Supplementary Figure 2 and Supplementary Table 2). Two *Pseudomonas* ASVs (ASV3, ASV4) showed abundances of >2% under control and BOA conditions, and two *Pseudomonas* ASVs (ASV2, ASV3) were also abundant under quercetin treatment. The *Pseudomonas* ASV4 showed a very high relative abundance of >20% at day 7 after BOA treatment and then declined, while it accumulated later in the control samples peaking at day 21 (16%). Two *Sphingobium* ASVs (ASV1, ASV2) very specifically increased under quercetin treatment, to 5.9 and 22.4% of relative abundance, respectively, while they were undetectable under all other conditions including control. One unclassified Micrococcaceae ASV increased to more than 30% after BOA treatment (Supplementary Figure 2B).

Taken together, treatment with BOA resulted in the increase in relative abundance of only ten ASVs, while gramine or quercetin treatment caused an increase in relative abundance of many more ASVs, particularly members of the Proteobacteria. On the other hand, all treatments caused a decrease in relative abundance of only few ASVs (Figure 5).

### Isolation of Cultivable Strains After Treatment of the Soil With Plant Metabolites

**FIGURE 6:**
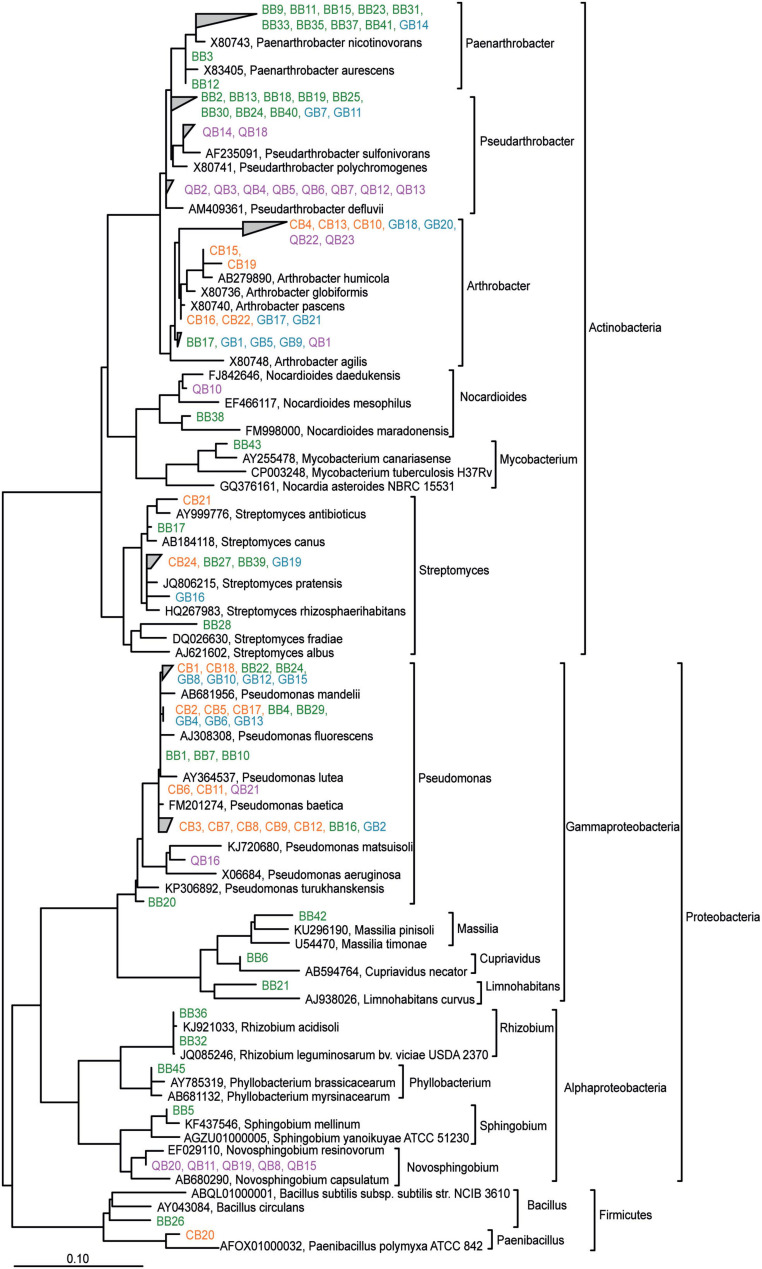
Phylogenetic relationship of bacterial isolates obtained after treatment of the soil with BOA, gramine or quercetin and from control soil. The tree was calculated based on the PhyML maximum-likelihood algorithm. Color coding indicates isolates from control soil or soils treated with BOA, gramine or quercetin. Type strain sequences are indicated in black.

To recover microorganisms that can withstand the treatment of the soil with the plant metabolites, microorganisms were isolated from the soil harvested after 28 days of incubation in the presence of BOA, gramine or quercetin and from control soil. The control soil was included to demonstrate that the identity of dominantly recovered isolates from metabolite-treated soils differs from that obtained from untreated soil. Isolates were characterized after enrichment and isolation on different complex media and DNA sequencing of the 16S *rRNA* gene. A total of 108 bacterial strains were isolated from control and metabolite-treated soil (Supplementary Table 3). We recovered Actinobacteriota (*Paenarthrobacter*, *Pseudarthrobacter, Arthrobacter, Nocardioides, Mycobacterium, Streptomyces*), Proteobacteria (*Pseudomonas, Massilia, Cupriavidus, Limnohabitans, Novosphignobium, Sphingobium, Rhizobium, Phyllobacterium*) and Firmicutes (*Bacillus*, *Paenibacillus*) from the treated or control soils. To identify the different isolates and assess the relationship between them, the 16S *rRNA* gene sequences were aligned and a phylogenetic tree was generated (Figure 6). From this analysis it became clear that many isolates can be divided into groups depending on the treatment. For example, one BOA-dependent *Paenarthrobacter* group, and two distinct groups of *Pseudarthrobacter* isolates from BOA or quercetin treated soil were identified (Figure 6). Further branches encompassed isolates from Burkholderiales (*Massilia, Cupriavidus, Limnohabitans*, isolated from BOA treated soil), a *Novosphingobium* branch dependent on quercetin treatment, *Sphingobium* (BOA dependent) and a *Rhizobiales* group (*Rhizobium*, *Phyllobacterium*, isolated after BOA treatment) (Figure 6). In contrast, closely related isolates of *Arthrobacter*, *Streptomyces*, and *Pseudomonas* were retrieved from two or more of the different treatments. Furthermore, the isolate sequences were aligned with those of related type strains and with ASV sequences obtained from soil DNA after treatment (Supplementary Figure 3). This analysis revealed that many isolated strains were related to specific ASV sequences, suggesting that these isolates represent the corresponding ASVs.

## Discussion

### General Effects of the Plant Metabolites on Soil Bacteria

Evidence is accumulating in recent years demonstrating that plant metabolites can modulate the profile of root-associated microbial communities (Stringlis et al., 2018; Huang et al., 2019; Voges et al., 2019; Harbort et al., 2020). The soil microbiota is crucial for agricultural productivity due to their contributions to and effects on nutrient mineralization, disease suppression, water retention or degradation of harmful molecules. A loss of species diversity and reduced abundance in the soil can therefore result in a plant microbiota being less rich in species diversity, resulting in decreased growth, increased susceptibility to diseases, and finally in harvest losses (Korenblum et al., 2020; Köberl et al., 2020). Agricultural practices such as continued monocultures without fallow periods can have detrimental effects on the soil microorganisms, and these effects can be caused by the release of plant metabolites into the soil (Li et al., 2019).

In the present study, we focused on the effects of two plant indole metabolites, BOA and gramine, mostly produced by Gramineae species, in comparison with the flavonoid quercetin, on soil bacterial communities. Bacterial 16S *rRNA* gene community profiling revealed that the three metabolites conveyed distinct effects on the community structure of soil-borne bacteria. These alterations are reflected by changes in alpha diversity after BOA and quercetin treatment, while alpha diversity of gramine treatment was not significantly different from control samples, when all time points of the treatment were aggregated (Figure 2). Treatment with the three metabolites caused alterations in taxonomic diversity as revealed in the PCA plot (Figure 3A). The data points for quercetin treatment were clearly separated from the control, in agreement with the differences observed for the alpha diversity indices (Figure 2). The early time points of the control were clearly separated from the BOA treatment data points, but the late control time points overlapped with those of the BOA treatment. However, aggregation of all time points in the PCA plot would result in a separation of the BOA and control treatment, which is in line with the differences in diversity indices (Figure 2). The design of the control experiment included the incubation of the soil without the addition of carbon sources. In analogy, treatment with BOA, which can be only poorly metabolized in the soil, mostly exerts inhibitory effects on bacterial growth. In the control experiment, easily accessible carbon initially present in the soil is presumably metabolized during the first 2 weeks, and thus the microbiota at later time points is more restricted in growth, resulting in changes in the community structure related to changes observed for BOA treated samples. The points of the gramine treatment, in particular the later time points, overlapped with the early control time points, which reflects the scenario that the diversity indices of control and gramine treatment were not significantly different (Figures 2, 3A). This result suggests that the diversity of the gramine treated samples remains high over time, resembling the one of early control time points, but is clearly different from the late control time points and those of BOA and gramine treated samples which strongly declined.

Microorganisms able to cope with the plant-derived compounds can remain in a metabolically active stage and may modulate soil parameters, such as pH, to their favor while shaping the species composition of other bacterial groups for optimization of co-existence and cooperation. Adapted microorganisms may group for acting in concert for compound detoxification and degradation (Korenblum et al., 2020; Pileggi et al., 2020). Microorganisms with these abilities obviously remain longer in the soil, even when the crop species change in subsequent cultures. These bacteria might give report about the history of soil usage, and about species particularly adapted to defined plant metabolites. PLFA analysis demonstrated that the treatment with plant metabolites did not affect the overall microbial biomass in the soil. Therefore, the alterations in the abundances of ASVs correlated with absolute changes in the amounts of the corresponding bacteria. Furthermore, the fatty acid composition indicated that the soil was dominated by a bacterial microbiota with very little fungal contribution. In parallel to the bacterial primer combination, attempts to amplify fungal sequences with a primer set specific to the fungal ITS sequences failed. These results indicate that the bulk of the Cologne agricultural soil contains very low amounts of fungi. As this soil was not used for agriculture for >15 years, it is presumably low in organic matter, which restricts the proliferation of fungi. In agreement with this scenario, fungal taxa have only been detected in the rhizosphere when this soil was used for plant growth experiments (Durán et al., 2018).

The effects of BX on bacteria in the rhizosphere were previously studied by growing BX-producing wild type (WT) or deficient (*bx*) maize plants in soil (Hu et al., 2018; Cotton et al., 2019; Kudjordjie et al., 2019). The relative abundance of specific bacterial taxa was decreased in WT compared with the *bx* mutants, and therefore was predominantly negatively affected by the presence of BXs in WT (Cotton et al., 2019; Kudjordjie et al., 2019). While it is known that the microbiomes of the rhizosphere and the bulk soil are essentially different because the rhizosphere microbiota represents only a low proportion of the bacterial biota in the bulk soil, this finding is in line with results obtained here after direct BOA application to the bulk soil, because only 10 ASVs increased in relative abundance, while 11 decreased (Figure 5). Because many soil-borne bacteria lacking rhizosphere competence might not be exposed to physiologically relevant concentrations of BOA by root exudation, but can get in contact with BOA by agricultural practices like crop rotation or mulches, the results obtained here are relevant for the evaluation of metabolite effects on soil quality. No effects of the plant genotypes of maize WT or *bx* mutants on the alpha diversity of the bacterial microbiota were detected (Hu et al., 2018; Cotton et al., 2019; Kudjordjie et al., 2019), in contrast to the present study where we treated bulk soil directly with BOA causing a decline in alpha diversity (Figure 1). These contrasting results might be caused by a different availability of BXs in the rhizosphere and bulk soil.

### Detection of Plant Metabolites After Addition to the Soil

The amounts of metabolites added to the soil in the present work (∼0.4 μmol per g soil) were in the range of the quantities used in previous studies. For example, BXs are released in high amounts (e.g., 0.5–5 kg/ha from field-grown rye) into the soil (Barnes and Putnam, 1987; Reberg-Horton et al., 2005). The amounts of BX in rye depend on the cultivar and the age of the plants, ranging from 160 to 2,000 μg/g dry matter. Understrup and coworkers concluded that amounts of 0.003, 3, and 30 μmol BOA/g soil are naturally reached by root exudation or decaying plant material (Understrup et al., 2005). The biotransformation of 30 μmol BOA/g soil was not completed after 90 days and even later, BOA was still detected. In phytotoxicity experiments, concentrations of 0.1–5.0 mM were used to study the effects of BOA on oxidative stress in mung bean (Batish et al., 2006). Highest amounts of gramine were found in young leaves of wild barley accessions, ranging from 2.032 to 5.290 μmol/g fresh weight, whereas the contents in roots were lower (Maver et al., 2020). Concentrations of 0.5 and 1 mM were employed to measure the phytotoxic effects of gramine on lettuce roots (Maver et al., 2020). Total quercetin in seeds of *L. japonicus* amounts to ∼1.4 mg/g (∼4.6 μmol/g) (Suzuki et al., 2008). Considering that quercetin can be released into the soil, local concentrations of ∼0.4 μmol/g soil might easily be reached. This concentration is in the same range as that of quercetin-glycoside released from white clover (0.5 μmol/g soil), and far below the amounts of luteolin, a flavonoid related to quercetin, released from peanut residues (0.42 μg/g, equivalent to 120 μmol/g soil) (Carlsen et al., 2012; Wang et al., 2018). In the present study, the three metabolites were added to the soil in total amounts ∼0.4 μmol/g of soil which is in the range of the naturally occurring concentrations.

HPLC measurements of the contents of BOA, gramine or quercetin in the soil showed that the metabolite contents were strongly decreased two days after the first application (Supplementary Figure 1). While BOA was completely extractable directly after addition to the soil, only 85% were recovered two days later. Therefore, BOA only weakly binds to soil particles, but is mostly degraded by the soil microbiota during the two days. In fact, a certain proportion of BOA is converted into APO in the soil, in line with previous results (Fomsgaard et al., 2004; Macías et al., 2005). BOA degradation results in the production of 2-aminophenol, which is dimerized to 2-aminophenoxazinone (APO). Therefore, in theory, one molecule of APO can be produced form two molecules of BOA. APO shows higher toxicity compared to BOA, and therefore, APO presumably is the more relevant compound affecting the soil microbiota. Some microorganisms, e.g., *Pseudomonas* species, convert 2-aminophenol into other metabolites further broken down in the TCA cycle, thereby diverting a considerable proportion of BOA degradation products away from APO production (Takenaka et al., 1998). In contrast to BOA, gramine and quercetin were strongly retained directly after addition to the soil, presumably binding to soil particles. Furthermore, the amounts of gramine and quercetin stayed low two days after addition. At the same time, low amounts of other unknown metabolites, which will be subject to structural elucidation in future studies, were found, indicating that gramine and quercetin were present in the soil and were broken down by the microbiota. Studies on the accumulation or degradation of gramine in soil or on the absorption to soil particles are not available. It is known that quercetin strongly interacts with and is adsorbed by soil particles (Terzano et al., 2015). In addition, quercetin is rapidly degraded by soil bacteria, e.g., *Pseudomonas putida* strains (Pillai and Swarup, 2002). These previous results are in line with the finding that quercetin was undetectable by HPLC in the soil samples (Supplementary Figure 1).

### Isolation of Bacterial Strains From Soil Treated With Plant Metabolites

One hundred seven bacterial isolates were obtained from the control or treated soil. The bacteria were isolated from one representative pot by plating a serial dilution of the soil extract on six different media. The media used for the growth of the individual bacterial isolates are indicated in Supplementary Table 3 and Figure 6, and it is possible that some of the isolates, in particular those with identical sequences, are clonal representatives of the same strain. A phylogenetic tree was built including the 16S rRNA sequences of the isolates, the closest type strains and the ASV sequences obtained from the soil DNA (Supplementary Figure 3) to unravel in which way the isolate sequences are related to bacterial type strains and the collection of ASVs. Indeed, for many isolates, related type strains or ASVs were found suggesting that they are related or even identical. For example, only one *Massilia* isolate, BB42, was found which is closely related to several cultivated *Massilia* species and even more similar to two neighbored ASVs (Supplementary Figure 3). In addition, BB42 is more distantly related to most other ASV sequences representing the genus *Massilia*. The finding that 38 ASVs, but only one isolate of the genus *Massilia* were retrieved, suggests that *Massilia* might be underrepresented in the isolation approach. The *Nocardioides* group with 65 ASVs, but only 2 isolate sequences, as well as the *Paenibacillus* and *Bacillus* groups, are further examples for genera that were presumably underrepresented in the isolate collection. In some cases, no related ASVs were found to be significantly affected by the treatment that was applied to obtain the isolate, underlining that cultivation-dependent methods are not necessarily in line with cultivation independent results. Many *Pseudomonas* isolates were obtained, some of which (e.g., BB1, GB12) were closely related to ASV sequences, and therefore presumably provide a good representation of these strains in the soil. Similarly, the group of *Pseudarthrobacter* encompassed 36 isolates but only 9 ASVs. While some of the isolates might be clonal (see above), this result still shows that a good representation of *Pseudarthrobacter* strains was isolated, and some of them are only distantly related to the ASV sequences suggesting that they represent bacterial species that were below the detection limit in the soil DNA sequencing. In the future, the isolates will be subject to detailed studies on the tolerance and toxicity for the 3 different metabolites.

### Specific Effects on Bacterial Microbiota in the Soil After Application of BOA, Gramine, or Quercetin

Application of BOA, gramine or quercetin to the soil resulted in the enrichment of *Paenarthrobacter*, *Pseudarthrobacter*, and *Arthrobacter* ASVs (Figure 5). *Pseudarthrobacter* and *Arthrobacter* were also frequently recovered isolates from treated soils (50 isolates of a total of 107) (Figure 6). The group of *Paenarthrobacter* contains 11 BOA-dependent and one gramine-dependent isolate. The first group of *Pseudarthrobacter* isolates encompassed two isolates each from quercetin or gramine treated soil, and 8 BOA-dependent isolates. The second *Pseudarthrobacter* group contained only eight isolates from quercetin treated soil. *Arthrobacter* and related *Actinobacteria* are wide-spread in bulk soil, are resistant to drying and starvation and can therefore live in extreme habitats (Knief et al., 2020). In this study, several ASVs related to xenobiotic-degrading strains increased in abundance or corresponding strains were isolated after metabolite treatment, for example *Paenarthrobacter* (Deutch et al., 2018). It is possible that these strains use related pathways to degrade plant metabolites including BOA, quercetin or gramine.

Several ASVs, including *Cupriavidus*, *Bosea*, *Allorhizobium*, and *Phyllobacterium*, the latter two representing members of the of the order *Rhizobiales*, were increased after treatment with BOA/quercetin, gramine or quercetin (Figure 5). Furthermore, three members of the *Rhizobiales* (two *Rhizobium*, one *Phyllobacterium* isolate) were obtained from the soil after BOA treatment (Figure 6). Some members of the Rhizobiales might belong the non-nitrogen fixing Rhizobia, of which many are members of the root microbiota (Garrido-Oter et al., 2018). Others might belong to the nodule-forming Rhizobia, which establish mutualistic interactions with legumes, thereby possibly contributing to increase the nitrogen availability in the soil with beneficial effects for the plants.

Furthermore, ASVs of *Bdellivibrio*, *Bacteriovorax*, and *Peredibacter* showed increased relative abundance after gramine or quercetin treatment, respectively. These bacteria of the Bdellovibrionota might be predators feeding on susceptible bacteria (Davidov et al., 2006). These taxa might also have been enriched by feeding on bacterial taxa that were stimulated in growth by the metabolites. It is tempting to speculate that members of *Bdellovibronota* were attracted by gramine or quercetin with the aim to eliminate hazardous or plant pathogenic bacteria, thus having beneficial effects for the plants.

Treatment with gramine or quercetin resulted in the increase in relative abundance of *Novosphingobium* and *Massilia* ASVs, and five *Novosphingobium* isolates were obtained after quercetin treatment. Specific strains of *Novosphingobium* or *Massilia* harbor plant growth promoting properties, e.g. auxin or siderophore production, and thus might also be beneficial for the growth of gramine- or quercetin-producing plants (Ofek et al., 2012; Rangjaroen et al., 2017).

Many *Pseudomonas* isolates were obtained (31 of 107 isolates) from control soil or after treatment with BOA, gramine, or quercetin (Figure 6), which might in part be due to the exceptional high growth rate of *Pseudomonas* strains. Only two *Pseudomonas* strains, QB16 and QB21, were found after quercetin treatment. A large branch contained 29 *Pseudomonas* isolates mostly from control, BOA-treated or gramine-treated soil. The abundance of the *Pseudomonas* ASVs was specifically decreased after gramine or quercetin treatment. The genus *Pseudomona*s encompasses many soil bacteria, some of which are plant pathogens, like *P. syringae.* It is possible that gramine or quercetin treatment in the present study decreased the abundance of pathogenic *Pseudomonas* strains, in agreement with previous results which showed that *P. syringae* is sensitive to gramine (Sepulveda and Corcuera, 1990). On the other hand, several *Pseudomonas* ASVs possibly with beneficial effects for the plants (Haas and Défago, 2005) were increased after BOA or quercetin treatment. BOA and quercetin application exerted considerable negative impacts on the relative abundance of bacterial taxa because 10 or 17 ASVs, respectively, decreased in relative abundance. The relative abundance of *Mycobacterium* was also decreased with BOA, in line with previous results which showed that isolates from these genera were sensitive to BXs (Anzai et al., 1960; Atwal et al., 1992; Schütz et al., 2019).

## Conclusion

Benzoxazolinone treatment caused the increase in the relative abundance of only 10 ASVs, while 11 ASVs of different phyla were inhibited. Therefore, BOA exerts a relatively strong negative effect on soil microbial diversity, with only few ASVs being able to proliferate. The general impact of the other two metabolites, gramine and quercetin, on soil bacteria, was related, because many more ASVs were increased after gramine (33) or quercetin (38) treatment, with most ASVs belonging to the Proteobacteria. On the other hand, 17 and 5 ASVs, respectively, were decreased under gramine or quercetin treatment. Therefore, BOA on the one hand and gramine/quercetin on the other hand reveal different effects on soil bacteria, with BOA showing a predominantly inhibitory effect preventing the accumulation of many presumably harmful taxa, while gramine and quercetin might mostly exert their function by attracting beneficial bacteria.

## Data Availability Statement

The datasets presented in this study can be found in online repositories. The names of the repository/repositories and accession number(s) can be found below: https://www.ncbi.nlm.nih.gov/, PRJNA699185; https://www.ncbi.nlm.nih.gov/, MW767719-MW767825.

## Author Contributions

VS, MS, and PD conceived the study. KF, PZ, SH, and PS-L contributed to the data analysis of bioinformatics. VS, JC, PZ, and SH contributed to the soil sampling. VS and JC performed DNA extractions and PLFA analysis. VS and PD contributed to draft the article. KF, PS-L, CK, MS, and PD contributed to critically review and edit the manuscript. All authors contributed to the article and approved the submitted version.

## Conflict of Interest

The authors declare that the research was conducted in the absence of any commercial or financial relationships that could be construed as a potential conflict of interest.

## Supplementary Material

Supplementary Figure 1 Contents of plant metabolites in the soil after treatment with BOA, gramine, or quercetin. Plant metabolites were mixed with 300 g of soil and after extraction with methanol, quantified by HPLC. Metabolites in the soil were measured directly after addition to record the proportion of the compounds absorbed to soil particles, or 2 days after addition to measure the amount of compounds degraded by soil microorganisms. Data show mean ± SD, n = 3; n.d., not detected.

Supplementary Figure 2 Alterations in relative abundances of individual ASVs during treatment with plant metabolites. The changes in relative abundances of eight highly abundant ASVs based on amplicon sequencing are shown for the different days of treatments with BOA, gramine or quercetin. **(A)** ASVs showing typical dynamics of responses to the different plant metabolites. **(B)** ASVs that showed strong differences in relative abundance in at least one soil treatment.

Supplementary Figure 3 Phylogenetic relationship of sequences from bacterial isolates and ASVs obtained after treatment of the soil with BOA, gramine or quercetin and from control soil, and with type strain sequences. The tree is based on the maximum-likelihood algorithm. Color coding indicates the different types of sequences from bacterial isolates (control, orange; BOA, green; gramine, blue; quercetin, red), ASVs (yellow) or type strains (black). The media used for isolation are indicated as A, PGA; B, TSB; C, Czapek; M, malt; T, TSM; Y, YPD.

Supplementary Table 1 Oligonucleotides used for PCR amplification and sequencing.

Supplementary Table 2 Responsive bacterial ASVs identified in the soil after treatment with plant metabolites.

Supplementary Table 3 Isolated e bacterial strains.
