# Supplementary material for: Differential Impact of Plant Secondary Metabolites on the Soil Microbiota
Source: Front Microbiol. 2021 May 28;12:666010. doi: 10.3389/fmicb.2021.666010 (PMC8195599; doi:10.3389/fmicb.2021.666010)
Supplement: Supplementary Figure 1 — Contents of plant metabolites in the soil after treatment with BOA, gramine, or quercetin. Plant metabolites were mixed with 300 g of soil and after extraction with methanol, quantified by HPLC. Metabolites in the soil were measured directly after addition to record the proportion of the compounds absorbed to soil particles, or 2 days after addition to measure the amount of compounds degraded by soil microorganisms. Data show mean ± SD, n = 3; n.d., not detected. [file Image_1.pdf]

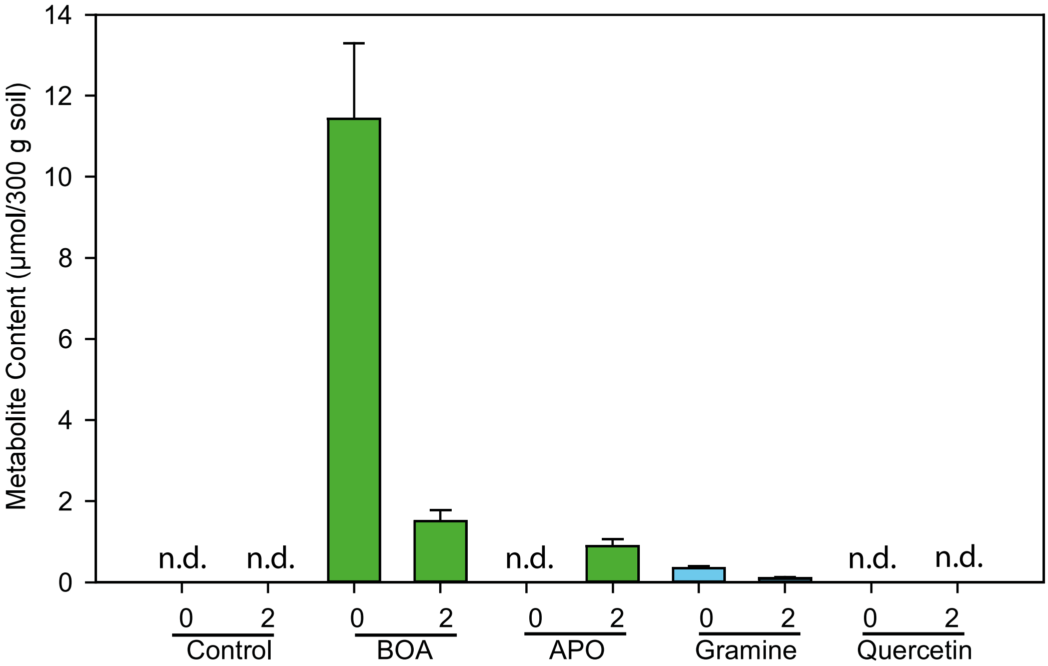

Figure S1. Contents of plant metabolites in the soil after treatment with BOA, gramine, or quercetin. Plant metabolites (10 µmol) were mixed with 300 g of soil and after extraction with acidic methanol, quantified by HPLC. The three metabolites and the BOA degradation product APO in the soil were measured directly after addition (0 days) to record the proportion of the compounds absorbed to soil particles, or 2 days after addition to measure the amount of compounds degraded by soil micro-organisms. Note that none of the metabolites was detected in the control samples. Data show mean ± SD, n=3; n.d., not detected.
