# Supplementary material for: Differential Impact of Plant Secondary Metabolites on the Soil Microbiota
Source: Front Microbiol. 2021 May 28;12:666010. doi: 10.3389/fmicb.2021.666010 (PMC8195599; doi:10.3389/fmicb.2021.666010)
Supplement: Supplementary Figure 2 — Alterations in relative abundances of individual ASVs during treatment with plant metabolites. The changes in relative abundances of eight highly abundant ASVs based on amplicon sequencing are shown for the different days of treatments with BOA, gramine or quercetin. (A) ASVs showing typical dynamics of responses to the different plant metabolites. (B) ASVs that showed strong differences in relative abundance in at least one soil treatment. [file Image_2.pdf]

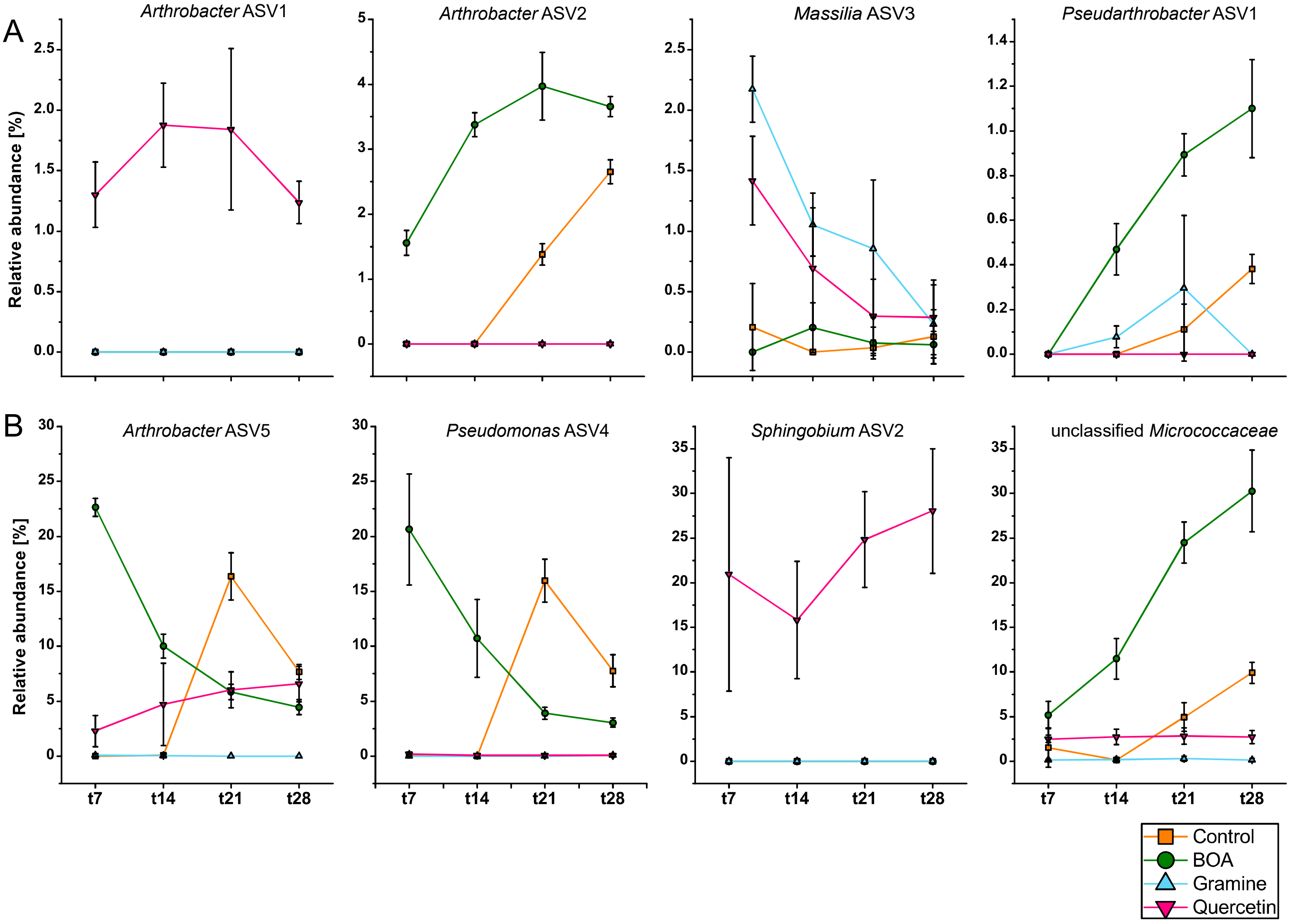

Figure S2. Alterations in relative abundances of individual ASVs during treatment with plant metabolites.

The changes in relative abundances of representative ASVs based on amplicon sequencing are shown for the different days of treatments with BOA, gramine or quercetin.

A, Representative ASVs showing typical dynamics of responses to the different plant metabolites.

B, Four ASVs that were highly abundant at least during one soil treatment.
