# Supplementary material for: Differential Impact of Plant Secondary Metabolites on the Soil Microbiota
Source: Front Microbiol. 2021 May 28;12:666010. doi: 10.3389/fmicb.2021.666010 (PMC8195599; doi:10.3389/fmicb.2021.666010)
Supplement: Supplementary Figure 3 — Phylogenetic relationship of sequences from bacterial isolates and ASVs obtained after treatment of the soil with BOA, gramine or quercetin and from control soil, and with type strain sequences. The tree is based on the maximum-likelihood algorithm. Color coding indicates the different types of sequences from bacterial isolates (control, orange; BOA, green; gramine, blue; quercetin, red), ASVs (yellow) or type strains (black). The media used for isolation are indicated as A, PGA; B, TSB; C, Czapek; M, malt; T, TSM; Y, YPD. [file Image_3.pdf]

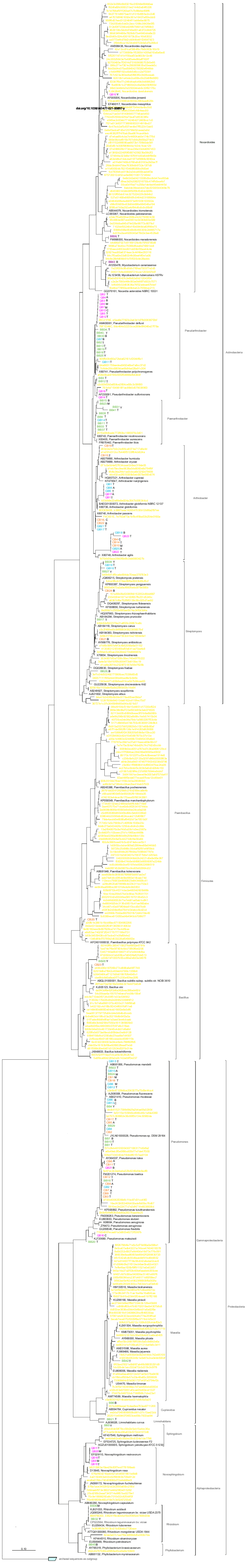

Figure S3. Phylogenetic relationship of sequences from bacterial isolates and ASVs obtained after treatment of the soil with BOA, gramine or quercetin and from control soil, and with type strain sequences.
The tree is based on the maximum-likelihood algorithm. Color coding indicates the different types of sequences from bacterial isolates (control, organge; BOA, green; gramine, blue; quercetin, red), ASVs (yellow) or type strains (black). The media used for isolation are indicated as A, PGA; B, TSB; C, Czapek; M, malt; T, TSM; Y, YPD.
