# Supplementary material for: Differential Impact of Plant Secondary Metabolites on the Soil Microbiota
Source: Front Microbiol. 2021 May 28;12:666010. doi: 10.3389/fmicb.2021.666010 (PMC8195599; doi:10.3389/fmicb.2021.666010)
Supplement: Supplementary Table 3 — Isolated e bacterial strains. [file Table_3.DOCX]

**Table S3. Isolated bacterial strains.**

| Isolate | Genus | Agar used for isolation |
| --- | --- | --- |
| Bacteria isolated from control soil | | |
| CB1 | *Pseudomonas* | Malt |
| CB2 | *Pseudomonas* | TSB |
| CB3 | *Pseudomonas* | TSM |
| CB4 | *Arthrobacter* | Czapek |
| CB5 | *Pseudomonas* | PGA |
| CB6 | *Pseudomonas* | PGA |
| CB7 | *Pseudomonas* | PGA |
| CB8 | *Pseudomonas* | PGA |
| CB9 | *Pseudomonas* | TSM |
| CB10 | *Arthrobacter* | Malt |
| CB11 | *Pseudomonas* | TSM |
| CB12 | *Pseudomonas* | TSM |
| CB14 | *Arthrobacter* | TSM |
| CB15 | *Arthrobacter* | TSM |
| CB16 | *Arthrobacter* | Czapek |
| CB17 | *Pseudomonas* | TSM |
| CB18 | *Pseudomonas* | TSM |
| CB19 | *Arthrobacter* | TSM |
| CB20 | *Paenibacillus* | TSM |
| CB21 | *Streptomyces* | TSB |
| CB22 | *Arthrobacter* | TSB |
| CB23 | *Bacillus* | TSM |
| CB24 | *Streptomyces* | TSB |
| Bacteria isolated from BOA treated soil | | |
| BB1 | *Pseudomonas* | TSM |
| BB2 | *Pseudarthrobacter* | TSM |
| BB3 | *Paenarthrobacter* | TSM |
| BB4 | *Pseudomonas* | YPD |
| BB5 | *Sphingobium* | YPD |
| BB6 | *Cupriavidus* | TSB |
| BB7 | *Pseudomonas* | TSB |
| BB9 | *Paenarthrobacter* | TSM |
| BB10 | *Pseudomonas* | TSM |
| BB11 | *Paenarthrobacter* | TSM |
| BB12 | *Paenarthrobacter* | TSM |
| BB13 | *Pseudarthrobacter* | TSM |
| BB15 | *Paenarthrobacter* | TSB |
| BB16 | *Pseudomonas* | TSM |
| BB17 | *Streptomyces* | TSM |
| BB18 | *Pseudarthrobacter* | TSM |
| BB19 | *Pseudarthrobacter* | TSM |
| BB20 | *Pseudomonas* | TSM |
| BB21 | *Limnohabitans* | TSM |
| BB22 | *Pseudomonas* | TSM |
| BB23 | *Paenarthrobacter* | Malt |
| BB24 | *Pseudomonas* | Malt |
| BB25 | *Pseudarthrobacter* | YPD |
| BB26 | *Bacillus* | YPD |
| BB27 | *Streptomyces* | YPD |
| BB28 | *Streptomyces* | TSB |
| BB29 | *Pseudomonas* | TSB |
| BB30 | *Pseudarthrobacter* | TSB |
| BB31 | *Paenarthrobacter* | YPD |
| BB32 | *Rhizobium* | YPD |
| BB33 | *Paenarthrobacter* | TSM |
| BB34 | *Pseudarthrobacter* | TSM |
| BB35 | *Paenarthrobacter* | TSM |
| BB36 | *Rhizobium* | Malt |
| BB37 | *Paenarthrobacter* | TSM |
| BB38 | *Nocardioides* | TSM |
| BB39 | *Streptomyces* | YPD |
| BB40 | *Pseudarthrobacter* | TSM |
| BB41 | *Paenarthrobacter* | TSM |
| BB42 | *Massilia* | TSM |
| BB43 | *Mycobacterium* | TSB |
| BB45 | *Phyllobacterium* | TSB |
| Bacteria isolated from gramine treated soil | | |
| GB1 | *Arthrobacter* | TSM |
| GB2 | *Pseudomonas* | TSM |
| GB4 | *Pseudomonas* | TSM |
| GB5 | *Arthrobacter* | TSM |
| GB6 | *Pseudomonas* | TSB |
| GB7 | *Pseudarthrobacter* | TSB |
| GB8 | *Pseudomonas* | Malt |
| GB9 | *Arthrobacter* | PGA |
| GB10 | *Pseudomonas* | PGA |
| GB11 | *Pseudarthrobacter* | TSM |
| GB12 | *Pseudomonas* | TSM |
| GB13 | *Pseudomonas* | Czapek |
| GB14 | *Paenarthrobacter* | TSM |
| GB15 | *Pseudomonas* | TSM |
| GB16 | *Streptomyces* | TSM |
| GB17 | *Arthrobacter* | TSM |
| GB18 | *Arthrobacter* | TSB |
| GB19 | *Streptomyces* | Malt |
| GB20 | *Arthrobacter* | PGA |
| GB21 | *Arthrobacter* | TSM |
| Bacteria isolated from quercetin treated soil (QB) | | |
| QB1 | *Arthrobacter* | TSM |
| QB2 | *Pseudarthrobacter* | TSM |
| QB3 | *Pseudarthrobacter* | TSB |
| QB4 | *Pseudarthrobacter* | PGA |
| QB5 | *Pseudarthrobacter* | TSM |
| QB6 | *Pseudarthrobacter* | Malt |
| QB7 | *Pseudarthrobacter* | Malt |
| QB8 | *Novosphingobium* | Malt |
| QB10 | *Nocardioides* | YPD |
| QB11 | *Novosphingobium* | TSM |
| QB12 | *Pseudarthrobacter* | TSM |
| QB13 | *Pseudarthrobacter* | Czapek |
| QB14 | *Pseudarthrobacter* | TSM |
| QB15 | *Novosphingobium* | TSM |
| QB16 | *Pseudomonas* | Czapek |
| QB17 | *Arthrobacter* | PGA |
| QB18 | *Pseudarthrobacter* | TSM |
| QB19 | *Novosphingobium* | TSM |
| QB20 | *Novosphingobium* | Malt |
| QB21 | *Pseudomonas* | TSB |
| QB22 | *Arthrobacter* | TSM |
| QB23 | *Arthrobacter* | TSM |
